# Monitoring Porcelain Insulator Condition Based on Leakage Current Characteristics

**DOI:** 10.3390/ma15186370

**Published:** 2022-09-14

**Authors:** Ali Ahmed Salem, Kwan Yiew Lau, Mohd Taufiq Ishak, Zulkurnain Abdul-Malek, Samir A. Al-Gailani, Salem Mgammal Al-Ameri, Ammar Mohammed, Abdulaziz Ali Saleh Alashbi, Sherif S. M. Ghoneim

**Affiliations:** 1 Institute of High Voltage and High Current, School of Electrical Engineering, Universiti Teknologi Malaysia, Johor Bahru 81310, Malaysia; 2 Faculty of Engineering, National Defence University of Malaysia (UPNM), Kuala Lumpur 57000, Malaysia; 3 School of Electrical and Electronic Engineering, Universiti Sains Malaysia, Nibong Tebal 14300, Malaysia; 4 School of Computing, Faculty of Engineering, Universiti Teknologi Malaysia, Johor Bahru 81310, Malaysia; 5 Electrical Engineering Department, College of Engineering, Taif University, Taif 21944, Saudi Arabia

**Keywords:** porcelain insulator, contamination, leakage current characteristics, classification

## Abstract

Insulator monitoring using leakage current characteristics is essential for predicting an insulator’s health. To evaluate the risk of flashover on the porcelain insulator using leakage current, experimental investigation of leakage current indices was carried out. In the first stage of the experiment, the effect of contamination, insoluble deposit density, wetting rate, and uneven distribution pollution were determined on the porcelain insulator under test. Then, based on the laboratory test results, leakage current information in time and frequency characteristics was extracted and employed as assessment indicators for the insulator’s health. Six indicators, namely, peak current indicator, phase shift indicator, slope indicator, crest factor indicator, total harmonic distortion indicator, and odd harmonics indicator, are introduced in this work. The obtained results indicated that the proposed indicators had a significant role in evaluating the insulator’s health. To evaluate the insulator’s health levels based on the extracted indicator values, this work presents the naïve Bayes technique for the classification and prediction of the insulator’s health. Finally, the confusion matrix for the experimental and prediction results for each indicator was established to determine the appropriateness of each indicator in determining the insulator’s health status.

## 1. Introduction

Outdoor insulators are important in electrical power transmission systems. However, the efficiency and health of the insulators are greatly impacted by numerous environmental factors, including wetness and contamination. Outdoor environmental factors, such as contamination and moisture, have a significant impact on insulator effectiveness. Moisture, such as rain and fog, in the presence of pollution reduces the surface resistance of insulators. Surface resistance lowering may result in higher leakage current flowing on the surface and dry band arcing. A large magnitude of leakage currents flowing on the surface over an extended length of time may produce insulator surface deterioration, which might eventually lead to flashover [1,2,3,4,5,6]. The unwanted discharge may result in a flashover phenomenon that leads to electrical grid disruption or even failure [7,8,9]. It is, therefore, crucial to monitor the status of the insulators to ensure that they are fit for purpose [10,11]. This would further strengthen the efficiency of the power grid and decrease its failure probability.

The evaluation of the performance of outdoor insulators continues to be a significant topic in the high-voltage community [12,13,14,15]. The use of the leakage current (LC) parameter in observing the performance of outdoor insulators has been popular and offers many advantages. This is because LC monitoring considers various environmental conditions, such as temperature, humidity, pollution, and rain [16]. Moreover, LC monitoring can be done online on a continuing basis. Examples of LC monitoring techniques include the use of the microwave reflectometer system, which has been used to monitor LC for dry insulator surfaces [17]. However, this type of monitoring system is less cost-effective. The authors in [18] offer an alternative method that uses an antenna to monitor LC on polluted insulators by capturing electromagnetic radiation resulting from partial discharge on the insulator. The advantage of this system is that the components used are not damaged by flashover voltage, as is the case of other systems. To the best of our knowledge, however, this system has yet to be tested under large electromagnetic interference caused by coronas and other effects of high-voltage conductors.

Another issue of interest in the monitoring technique of polluted insulator LC refers to the competence to establish a strong link between the LC and the condition of the insulator under service. Many researchers have offered various approaches to evaluate the physical state of insulators [16,19,20,21]. Improvement in LC-based monitoring is achieved by extracting information from the LC components. The LC statistical results, such as average, maximum, and standard deviation, recommended by [22] were used for evaluating the level of contamination. These researchers suggested that these parameters allow the quantification of the dimensions and density of the contamination layer over the surface of the insulator. Another study [19] assessed the contaminated insulator conditions by evaluating the phase angle between current and voltage signals. According to the results in [19], phase angle differences are a helpful indication for assessing contaminants and humidity fluctuations in a clean environment.

The assessment of the LC signal in the frequency domain through fast Fourier transform (FFT) and wavelet transformations is also a relevant technique used to predict insulators’ pollution status [23,24,25]. Overall, the results have indicated that contaminants on the insulator amplify the harmonic of leakage current components, especially the harmonics in an odd order. The results imply that the contamination leads to the rise of the first and third harmonics and the total harmonic distortion (THD) [26]. The concerned harmonics are the first and third component harmonics under an AC voltage. Accordingly, the study found that increasing these harmonics causes a rise in THD, which changes according to the change in the level of contamination and harmonics of the supplied voltage [27].

Some publications in the literature have proposed several numerical indices to identify the performance of overhead line insulators. As far as we are aware, none of them evaluated the performance of insulators using one specific indicator. In direct connection, acoustic and thermal-based diagnostic methods, such as ultrasonic wave [28] and acoustic fault diagnosis [29], have been used to evaluate the health of overhead lines insulator. However, the performance of these techniques is affected by the acoustic frequency noise generated from the electromagnetic field of the overhead line. Infrared thermal imaging [30] and temperature analysis of infrared insulator images [31] are utilized to diagnose polluted insulators as well. However, these techniques rely only on the thermal behavior of the insulator, which is limited in revealing an accurate insulator state. Although the wireless-based system [32] and network sensors [18] show good performance in monitoring insulator conditions, their high sensitivity to environmental weather, such as dust and rain, influences the accuracy of the results.

It is essential to have an indicator reflecting the condition of the insulators [16,20]. The extraction of the LC components based on the frequency domain to calculate the relevant indicators were performed. For example, the ratio of leakage current’s third to fifth harmonic indicator (fifth/third) was extracted to estimate flashover incidences [27]. The published findings for silicon rubber and glass insulators show good correlation between the magnitude of the contamination and the reading of this indicator. In addition, the literature review demonstrated that no attempt has been made to investigate the insulators’ conditions using indices considering the slope of the signal in the time domain and the odd harmonic components between 0 and 500 Hz for LC. This technique is supposed to yield a more reliable prediction when compared with the other leakage current indicators.

Regarding the classification and prediction of insulator health, the previous work in [33] introduces the support vector machine (SVM) for classifying the condition of the insulator under the effect of the different coating damage modes. The assessment of the contaminated insulators was performed using computer vision in [34] and k-nearest neighbors (k-NN) [35]. The results of these studies indicated that these techniques provide adequate efficiency and accuracy and that they are promising approaches since they are quick and easy to perform. Some researchers have evaluated the conditions of insulators using different artificial intelligence methods, as reported above. However, the application of the naïve Bayes method to estimate the insulator conditions based on the leakage current characteristics has not been investigated, and this will be discussed in our current work.

Our current work contributes to estimating the conditions of porcelain insulators, as follows:

- Six indicators based on the measured LC characteristics in the time domain and frequency domain, namely, the peak current indicator, phase shift indicator, slope indicator, crest factor indicator, total harmonic distortion indicator, and odd harmonic indicator, were extracted by taking into consideration the significant effect of environmental factors on the performance of the overhead line insulators. Environmental factors, including the soluble deposit density, wetting rate, insoluble deposit density, and contamination ratio of the upper to the lower side of the insulator were taken into consideration while simulating the nature of insulators in service.
- The classification of the state of insulators based on the proposed indicator values using the naïve Bayes approach was conducted.
- The comparison of the performance of the proposed indicators using the confusion matrix for the actual insulator conditions and naïve Bayes prediction results was carried out.

The rest of this paper is structured as follows. Section 2 reviews the process of pollution of the insulators and discusses how LC is measured. Section 3 illustrates the proposed leakage current indicator expressions. Section 4 presents the experimental and modeling results. Finally, the conclusion is presented in Section 5.

The advantages of this study are:

- The proposed indicators are useful to monitor the condition of overhead line insulators in real time.
- Insulator condition estimation using LC indicators is simple, low cost, and accurate.
- Applicable for any insulator type and any voltage level.
- Monitoring insulator conditions on the transmission line (without removing the insulator and without interrupting the power line).

## 2. Materials and Methods

### 2.1. Test Sample

**Figure 1:**
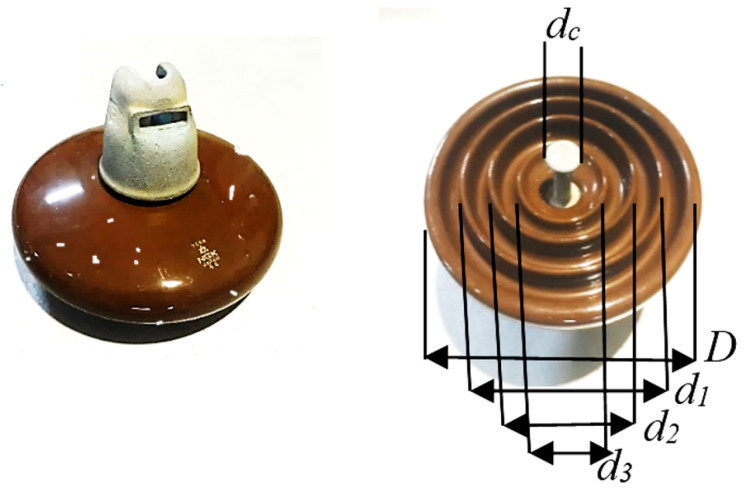
Insulator sample.

The porcelain insulators to be tested were collected from the transmission division of the national network in Malaysia. The selected insulators’ main shape is portrayed in Figure 1. The insulators’ actual specifications are tabulated as in Table 1. In this paper, a single disk of porcelain insulators and a string of three units of porcelain insulators were tested.

### 2.2. Test Facilities and Procedures

**Figure 2:**
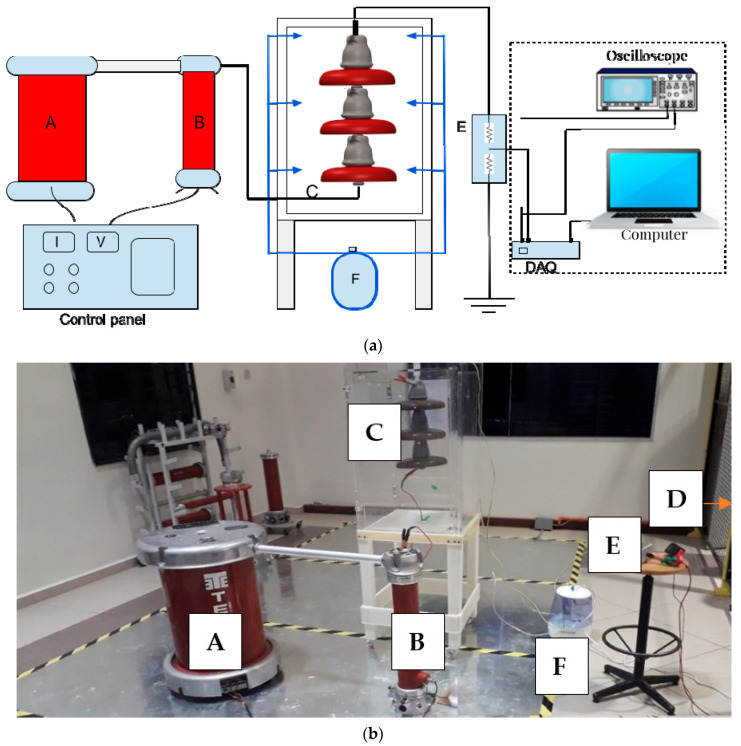
(**a**) Diagram of insulators’ test arrangement; (**b**) photograph of the insulators under test.

The IEC-507 standard, namely, “Artificial pollution tests on high-voltage ceramic and glass insulators to be used on AC systems” [36] was referred to while performing the experimental setup. All experiments were carried out in an artificial test chamber with dimensions 50 cm × 50 cm × 75 cm in which the walls were polycarbonate sheets. The chamber was installed with four inlet valves used for spraying insulators under test for the purpose of wetting. Figure 2a shows a schematic diagram for the high-voltage polluted insulator experimental setup. A photograph of the test setup and the equipment used in the high-voltage laboratory is given in Figure 2b. The experimental circuit setup was composed of the following components: A is a transformer (220 V/100 kV, 5 kVA, 50 Hz), B is a capacitive voltage divider, C is the test sample inside the chamber, D is a monitoring system to measure the leakage current, E is a resistive step-down divider (100:1) employed for the measurement of the LC and protection of the DAQ device, and F is a fog generator with a rate controller for wetting.

### 2.3. Wetting and Polluting

Prior to commencing the experiment, traces of grease and dirt were removed carefully for all specimens using alcohol liquid. Next, the insulators were dried for 24 h naturally. The contamination was then applied to the insulator surface based on the solid layer method [37,38,39,40]. The pollution was made up of two types of deposits: soluble deposit density (SDD), which is represented by sodium chloride salt (NaCl), and insoluble deposit density (NSDD), which was represented by kaolin. To prepare the SDD, the required amount of NaCl salt was mixed with 1000 mg of water to establish the pollution solution. Then, a conductivity meter was used to measure the conductivity of the polluted solution σ_σ_ at room temperature for three amounts of salt (10 g, 20 g, and 30 g) to compute three levels of SDD. Also, three amounts of kaolin (20 g, 40 g, and 60 g) were used to produce the NSDD levels listed in Table 2. Next, the conductivity of the pollution solution at 20 °C was calculated using Equation (1):

(1) $$\sigma_{20}=\sigma_{\sigma }\times \left[1-b\left(\theta -20\right)\right]$$

where $\sigma_{20}$ and $\sigma_{\sigma }$, represent the solution conductivity at 20 °C, and the solution conductivity at the room temperature. *θ* is the temperature of the solution and *b* is a temperature-dependent factor that is determined to be 0.02 at = 27.5 °C using Equation (2) [36].

(2) $$b=-3.2\times 10^{-8}\theta^{3}+1.032\times 10^{-5}\theta^{2}-8.272\times 10^{-4}\theta +3.544\times 10^{-2}$$

The SDD was calculated based on the IEC 60507 [36] and IEC 60815 [41] standards using Equation (3):

(3) $$SDD=\frac{{\left(5.7\times \sigma_{20}\right)}^{1.03}\times V}{S}$$

where *V* and *S* represent the volume of pollution solution, and the insulator surface area, respectively. Meanwhile, Equation (4) was used to calculate NSDD according to the IEC-507 standard [36]:

(4) $$NSDD=\frac{\left[(w_{s}-w_{i})\times 10^{3}\right]}{S}$$

where $w_{s}$ and $w_{i}$ are the mass of the filter paper under contamination and under dry conditions, respectively.

As shown in Table 2, three levels were determined for both SSD and NSDD: light, moderate, and high contamination. The sample was then polluted and hung in the test room and left drying for around 1 day to ensure that the polluted insulator was dried. The test room pressure remained constant throughout the experiment, matching the laboratory’s ambient pressure. The temperature in the testing room was roughly 27.9 °C, which was about the same as the indoor temperature in the laboratory. The relative humidity inside the test room was set to 75% and monitored during the testing with the help of a humidity sensor. The spray technique was used to wet the insulator, with six nozzles regularly spaced around the test room wall. A control panel outside the test room was used to automatically calculate the fog flow rate. To achieve the moisture of insulators at different levels, three degrees of wetting rates Wt were applied, i.e., 3 mL/h, 6 mL/h, and 9 mL/h.

Under both uniform and uneven contamination distributions, the porcelain was investigated. Also, three contamination ratios of the upper to the lower side of the insulator $\left(P_{L}/P_{u}\right)$, i.e., 1/3, 1/5, and 1/8, were selected in the uneven contamination case. The upper and lower surfaces of the insulator were polluted separately in the nonuniform pollution case to yield *SDD_u_* and *SDD_L_*, whereas the overall SDD can be met by Equation (5) [5,40]:

(5) $$SDD=\frac{SDD_{u}\times S_{u}+SDD_{L}\times S_{L}}{S_{u}+S_{L}}$$

where $S_{u}$ and $S_{L}$ are the area of the upper and lower surface of insulator, respectively. According to these selected pollution ratios, the SDD of the upper and lower sides ($SDD_{u}$ and $SDD_{u}$) can be satisfied by Equation (6):

(6) $$SDD_{Pu}=\frac{2\times SDD}{1+(P_{L}/P_{u})},\;SDD_{P_{L}}=\frac{2\times SDD}{1+(P_{u}/P_{L})}$$

### 2.4. Data Monitoring

As illustrated in the experimental setup in Figure 2, the input voltage values were obtained using a divider consisting of two capacitors with a ratio (C_1_ to C_2_) of 10,000:1. On the other side of the test chamber, the monitoring system consisted of a data acquisition (DAQ) card, oscilloscope, and a PC, which was used to measure LC. A resistive divider (100:1) was employed, since the DAQ’s input voltage range is just 10 V. During the measurement, the LC data were captured from the divider and then transferred using a DAQ card to the PC. The captured data were saved in a CSV file after being displayed on a graphical user interface of the LabVIEW software. For measurement validation, the oscilloscope was utilized to compare with DAQ data reading. Finally, with the help of MATLAB software, the LC data saved were converted into the frequency domain using FFT.

## 3. Characteristic Parameter of Leakage Current

In this paper, six characteristics/indices were extracted in both the time and frequency domains of LC to predict the health of polluted insulators.

### 3.1. Leakage Current Indicators in Time Domain

The time and frequency domain signal of LC was used to derive all the leakage current indicator equations. The formulae presented in this section were used as the indicators of change in LC signal that was affected by the insulator conditions. In the time domain of LC, four indicators were selected. The first and second indicators, which were the peak of leakage current *I_m_* (denoted as *x*_1_), found based on the absolute value of the current signal, and the phase shift ϕ between the applied voltage and LC (denoted as *x*_2_) were extracted from the LC general equation, expressed as in Equation (7) [42]:

(7) $$I=I_{m}\sin(\omega t+\phi )$$

where *ω* is the angular frequency equivalent to 2*πf*, with the value of frequency *f* in this study 50 Hz. As such, the first two characteristics can be defined as in Equations (8) and (9):

(8) $$x_{1}=I_{m}$$

(9) $$x_{2}=\phi =\frac{\Delta t}{T}360{}^{\circ}$$

**Figure 3:**
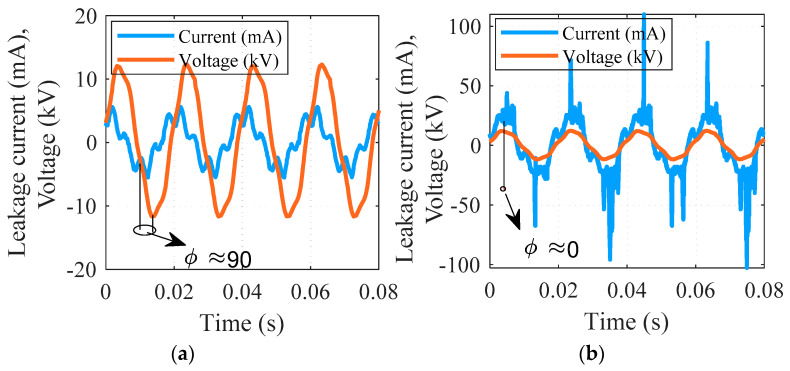
Leakage current and applied voltage phase angle: (**a**) clean; (**b**) pollution.

Figure 3 illustrates how the phase difference *ϕ* between the applied voltage and LC can be determined for a clean and a polluted insulator. For a clean insulator, the phase difference *ϕ* between the applied voltage and LC appeared to be nearly 90° (see Figure 3a). As the amount of pollution on the insulator increased, the phase difference *ϕ* between the applied voltage and LC decreased. Under heavily polluted circumstances, the phase difference *ϕ* between the applied voltage and LC became nearly zero (see Figure 3b).

**Figure 4:**
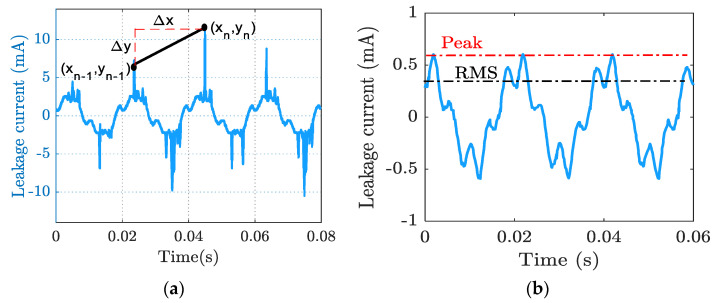
(**a**) Slope curve extracted from leakage current waveform; (**b**) crest factor indicator extracted from leakage current waveform.

The third indicator *x*_3_ was extracted by calculating the slope of the line between two consecutive peaks of LC signal, expressed as in Equation (10):

(10) $$x_{3}=\frac{\sum_{n=1}^{m}\left|y_{n}-y_{n-1}\right|}{x_{n}-x_{n-1}}=\frac{\sum_{0}^{m}\left|\Delta y_{n}\right|}{\Delta x_{n}}$$

where ∆*y_n_* is the LC difference for adjacent peaks at the nth point for time and ∆*x_n_* is the time between these peaks. Figure 4a illustrates how the LC signal slope was calculated. The fourth indicator *x_4_* was obtained from the crest factor, which was calculated by dividing the peak value with the RMS value of the LC waveform, as shown in Figure 4b. As such, *x_4_* is expressed as in Equation (11):

(11) $$x_{4}=\frac{I_{peak}}{I_{RMS}}$$

(12) $$I_{RMS}=\sqrt{\frac{1}{n}\sum_{i}i_{i}^{2}}$$

where RMS is root mean square, *i_i_* is each measured value, and *n* is the number of measurements.

### 3.2. Leakage Current Indicators in Frequency Domain

The frequency-domain signals of LC for polluted insulators have characteristic features at frequencies below 500 Hz. In this paper, the odd harmonic and total harmonic distortion (THD) of LC under 500 Hz were used to propose indicators for insulator condition assessment. The frequency characteristics of the LC are defined by the *x*_5_ and *x*_6_ indicators, as in Equations (13) and (14), respectively:

(13) $$x_{5}=THD=\frac{\sqrt{\sum_{n=2}^{\infty }I_{n}{}^{2}}}{I_{1}}$$

(14) $$x_{6}=\frac{\sum_{n}I_{n}}{I_{3}}\;\;\;\;n=5,7,9$$

where *I_n_* represent the *n*th harmonic and *n* is the odd-order harmonic number.

## 4. Classification Model

**Figure 5:**
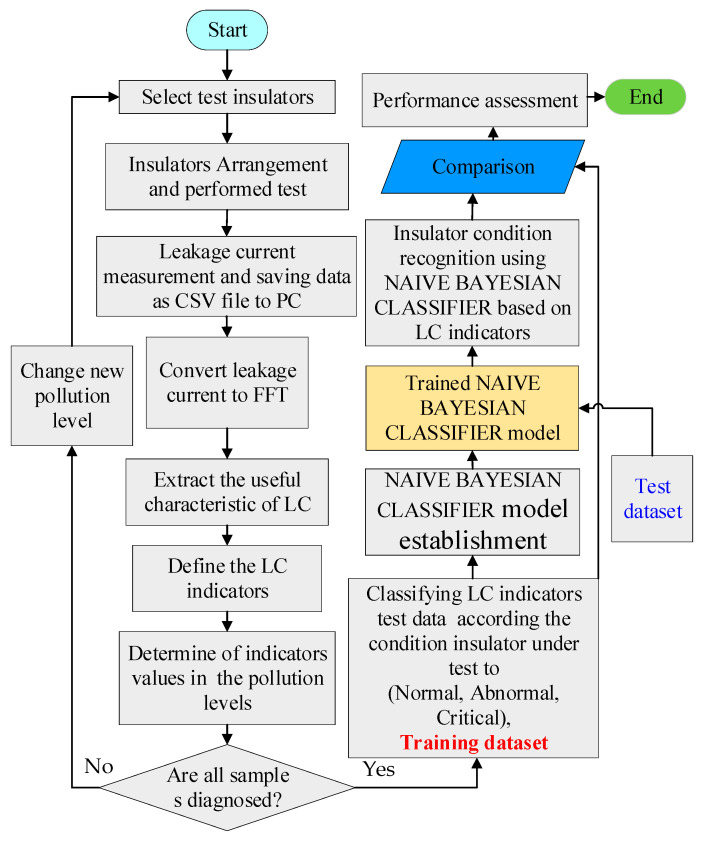
Data extraction and classification of the proposed indicators.

The naïve Bayesian classifier is a classification algorithm based on Bayes’s theorem. Its underlying idea and building approach are more straightforward and simpler than those of support vector machines and neural networks. Furthermore, compared to other algorithms, the naïve Bayesian classifier algorithm suits the approach of this work, and brought great accuracy in classifying the insulator situations. The naïve Bayesian classifier theorem, which is employed in the classification of the suggested LC indicators, is explained in this section. Figure 5 depicts the flowchart of the procedures involved in extracting and categorizing the recommended indicators.

### Naïve Bayesian Classifier

To understand the naïve Bayesian method, consider *y* to be a collection of samples. Each sample contains *n* condition characteristics that represent its special traits, as well as one class label. All LC features are assumed to be interrelated in this work, while the class label is assumed to be separated. Assume that all training examples are classified into *m* classifications and that the class label of each sample changes from {*z*_1_, *z*_2_, …, *z_m_*}. As a result, any sample can be shown as an *n*-dimensional vector. $\vec{y}$ = (*y*_1_, *y*_2_, …, *y_n_*) would be a piece of testing data whose class label has to be determined. A naïve Bayesian classifier may compute the posterior probabilities and decide the class label for the new sample based on the previous and class-conditional possibilities of the new sample. A naïve Bayesian classifier uses Equation (15) to characterize the new sample’s class label [43]:

(15) $$\begin{array}{l} z=\underset{z_{k},k=1,2,\ldots ,m}{\arg\max}\{P(z_{k}|\vec{y})\}\\ =\underset{z_{k},k=1,2,\ldots ,m}{\arg\max}\left\{\frac{P(z_{k})P(\vec{y|}z_{k})}{P(\vec{y)}}\right\}\\ =\underset{z_{k},k=1,2,\ldots ,m}{\arg\max}\left\{P(z_{k})P(\vec{y|}z_{k})\right\} \end{array}$$

where $P(z_{k})$ represents the previous probability of the *z_k_* class that can be found from $P(z_{k})=N_{k}/N$, *N_k_* is the number of samples within *z_k_* class, *N* is data set size, and $P(\vec{y|}z_{k})$ represent the class-conditional probability. The main aim of the naïve Bayesian classifier is to determine $P(\vec{y|}z_{k})$ based on the training samples in the *z_k_* class.

All features are assumed to be independent by the naïve Bayesian classifier. As a result, the class-conditional probability can be written as in Equation (16):

(16) $$P(\vec{y|}z_{k})=P(y_{1},y_{2},\ldots ,y_{n}|z_{k})=\prod_{i=1}^{f}P(y_{f}|z_{k})$$

To find the class label of $\vec{y}$, the Naïve Bayesian classifier can substitute the class-conditional probability with Equation (16) and yield the decision function in Equation (17).

(17) $$z=\underset{z_{k},k=1,2,\ldots ,m}{\arg\max}\left\{\frac{n_{k}}{N}\prod_{i=1}^{f}P(y_{f}|z_{k})\right\}$$

where $P(y_{f}|z_{k})(1\leq f\leq n)$ is an essential factor in determining the class label of the new sample.

## 5. Results and Discussion

### 5.1. Leakage Current Results

**Figure 6:**
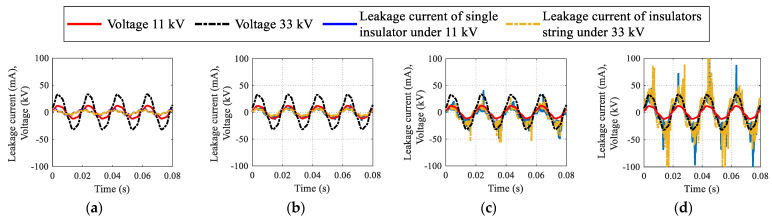
Leakage current waveform under NSDD = 0.15 mg/cm^2^, wt = 3 mL/h and different SDD: (**a**) SDD = 0.00 mg/cm^2^; (**b**) SDD = 0.05 mg/cm^2^, (**c**) SDD = 0.12 mg/cm^2^, (**d**) SDD = 0.2 mg/cm^2^.

**Figure 7:**
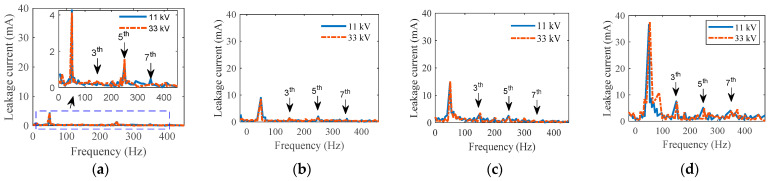
FFT of leakage current waveform under NSDD = 0.15 mg/cm^2^, Wt = 3 mL/h and different SDD: (**a**) SDD = 0.00 mg/cm^2^; (**b**) SDD = 0.05 mg/cm^2^, (**c**) SDD = 0.12 mg/cm^2^, (**d**) SDD = 0.2 mg/cm^2^.

**Figure 8:**
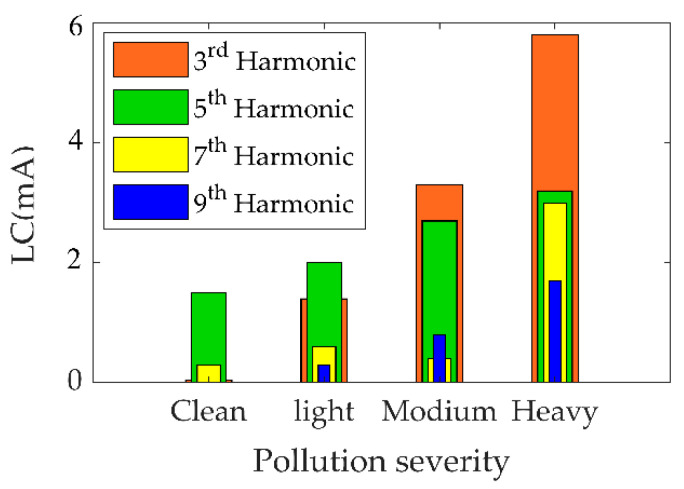
Odd harmonics of leakage current under pollution grading [2].

The LC findings of uniformly polluted insulators in both time and frequency domains with various SDD levels but fixed NSDD of 0.15 mg/cm^2^ and Wt of 3 mL/h are shown in Figure 6 and Figure 7. Figure 6 indicates that increased pollution severity causes a considerable rise in LC under specific values of NSDD, Wt, and P_u_/P_L_. The LC increase can be explained by the increase in conductivity of the pollution layer on the insulator’s surface once subjected to wetness. Consequently, the LC flowed from the high voltage terminal to the ground in the form of positively and negatively charged ions. Some spot arcs were observed on occasion under high-contamination conditions, particularly in the existence of moisture. During the flashover, the signal of the LC appeared to be severely distorted, as shown in Figure 6d. Furthermore, when LC increased, the THD and harmonic values increased (see Figure 7), while the phase angle between the current and voltage decreased. The decrease in phase angle between the LC and voltage is due to the resistive current increasing with constant capacitive current. Once the contamination level on the insulator surface was raised, a clear change in the odd harmonics (3rd, 5th, 7th, and 9th) was observed (see Figure 7). As seen in Figure 8, the 3rd harmonic will increase to surpass the 5th, 7th, and 9th harmonics, with a clear increase in the 7th and 9th harmonics. Furthermore, during discharge activity on the insulator’s surface, the 3rd harmonic is often substantially high [44,45].

Table 3 provides the test results of the LC harmonic components’ values (magnitude *I_m_*, harmonics, THD, and phase shift angle *ϕ*) for different pollution levels under uniform pollution distribution for all investigated conditions. Under the clean condition, the 5th and 7th harmonics were greater than the 3rd harmonic. Furthermore, there were no signs of flashover. The LC rose marginally as the wetting rate increased when the clean insulators were tested under a specific NSDD. This indicates that wetting the insulator surface caused the flow of the charges from the high-voltage terminal to the ground to rise noticeably.

Table 3 also shows the results of the uniformly polluted insulators with the change in SDD, Wt, and NSDD. Table 3 shows that the LC on a clean insulator’s surface is very low, approximately 1.81 mA for single disk and 1.78 for string insulators. This indicates that under clean circumstances, the performance of the insulators is capacitive. Because of the capacitive property of LC, the phase shift angle between current and voltage will be about 90°. Under the clean and dry scenario, the 5th harmonic is higher than the 3rd harmonic. Generally, the LC component test results in Table 3 show that:

1. Under dry conditions, surface conductivity was minimal. Therefore, the influence of increasing SDD and NSDD on LC and LC characteristics in this condition was minor.
2. The LC magnitude grew substantially as the contamination severity of SDD, NSDD, and Wt increased.
3. As SDD, NSDD, and Wt increased and P_u_/P_L_ decreased, odd harmonic values and THD increased. In contrast, the phase angle decreased.
4. When the Wt was changed for a clean insulator under a specific NSDD, the LC value varies somewhat, as do the temporal and frequency characteristics of the LC.

### 5.2. Leakage Current Indices Finding

**Figure 9:**
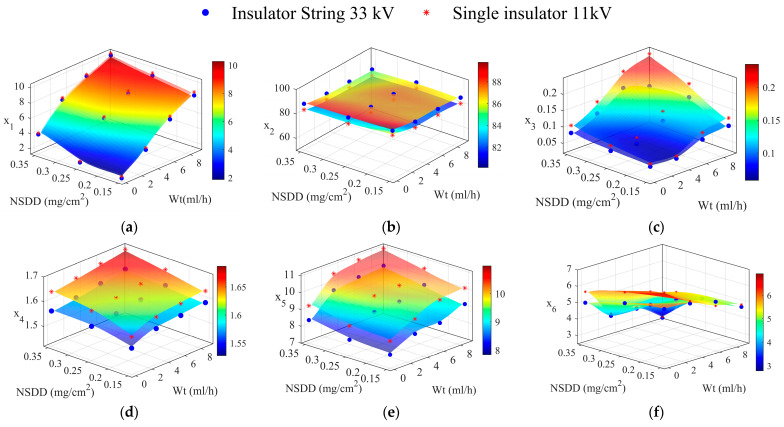
LC indices of a clean insulator under different Wt and NSDD; (**a**) x_1_; (**b**) x_2_; (**c**) x_3_; (**d**) x_4_; (**e**) x_5_; (**f**) x_6_.

Figure 9 shows the LC indices of a clean (0.00 mg/cm^2^ of SDD) insulator under different Wt and NSDD. Each indicator demonstrates a unique behavior when the wetting rate and NSDD were changed. Of note, there is no significant difference between different NSDD under the same Wt. The x_1_, x_3_, x_4_, and x_5_ indices increased with the rise in both the NSDD and Wt. For example, when the Wt was increased from 3 mL/h to 9 mL/h under 0.15 mg/cm^2^ of NSDD, the x_1_ increased from 4.2 to 8.88, x_3_ increased from 0.065 to 0.11, x_4_ increased from 1.56 to 1.585, and x_5_ increased from 7.6 to 10.49.

In contrast, the x_2_ and x_6_ decreased with the increase in both the Wt and NSDD. For example, when the Wt increased from 3 mL/h to 9 mL/h under NSDD = 0.15 mg/cm^2^, the x_2_ decreased from 87.2 to 86.01 and x_6_ decreased from 5.9 to 4.7.

#### 5.2.1. Indicators Trends under Different SDD

**Figure 10:**
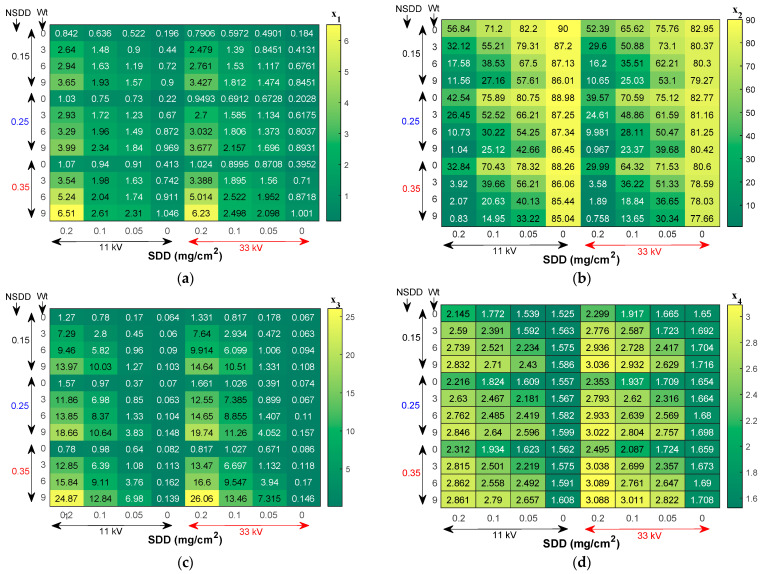

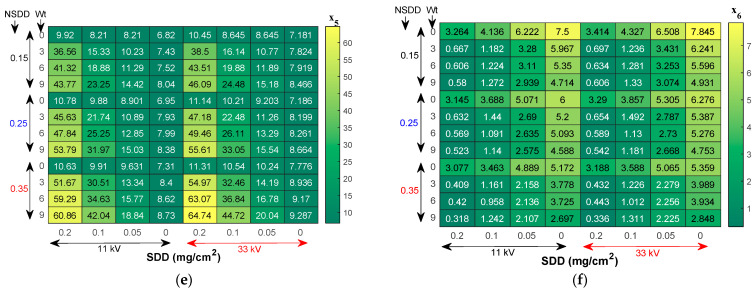
Leakage current indices of uniform polluted insulators under various wetting rate Wt and NSDD: (**a**) x_1_; (**b**) x_2_; (**c**) x_3_; (**d**) x_4_; (**e**) x_5_; (**f**) x_6_**.**

The leakage current indices x_1_, x_3_, x_4_, and x_5_ under different SDD, NSDD, Wt, and P_u_/P_L_ for single and string insulators are presented in Figure 10 and Table 4. The LC indicators of insulators under test increased with the increase in SDD under specific NSDD, Wt, and P_u_/P_L_. On the contrary, the indices x_2_ and x_6_ of insulators decreased with the increase in SDD under the same conditions. For example, under NSDD of 0.25 mg/cm^2^, Wt of 6 mL/h and P_u_/P_L_ of 1/3, when SDD was 0.05, 0.1 and 0.2 mg/cm^2^, the x_1_ for the single disk insulator corresponded to 12.2, 15.4, and 26.6 mA, respectively. The x_1_ also increased by 26.2% and 118.03% when the SDD increased from 0.05 to 0.1 mg/cm^2^ and from 0.1 to 0.2 mg/cm^2^, respectively. For x_6_, when the SDD was 0.05, 0.1 and 0.2 mg/cm^2^, x_6_ corresponded to 2.69, 1.31, and 0.63 mA, respectively. The LC indicators showed a similar trend and performance for the insulator string under 33 kV with minor variations, as illustrated in Table 5.

#### 5.2.2. Indicator Trends under Different NSDD

**Figure 11:**
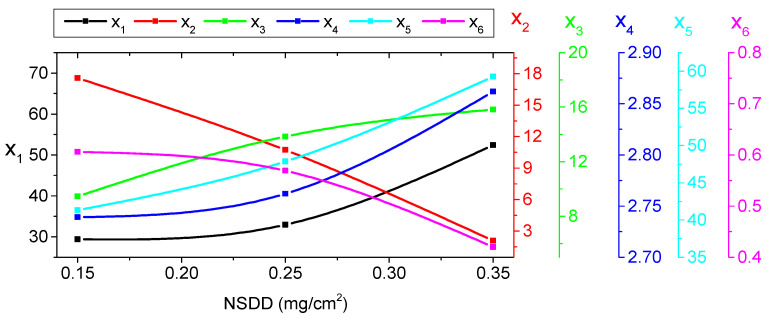
The impact of NSDD on indicator shift.

The differences in the indicators are comparable to the previous case (pollution variation), and changes in the amount of increment/or decrement can be detected. The test findings in Table 5 demonstrate that under constant SDD, Wt, and Pu/PL, increasing the NSDD increases the x_1_, x_3_, x_4_, and x_5_, but decreases the x_2_ and x_6_. To further understand the relationship between NSDD and the suggested indices, Figure 11 displays the x_1_, x_3_, x_4_, x_5_, and x_6_ vs. NSDD curves with SDD of 0.2 mg/cm^2^, Wt of 6 mL/h, and P_u_/P_L_ of 1/1.

#### 5.2.3. Indicator Trends under Different Wt

**Figure 12:**
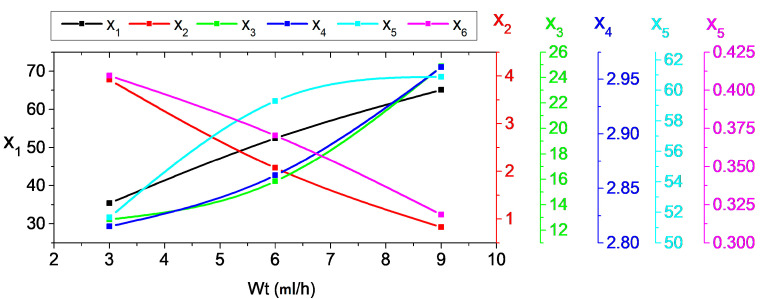
The impact of Wt on indicators shift.

The relationship between the proposed indices x_1_–x_6_ and Wt for porcelain insulator under SDD of 0.2 mg/cm^2^, NSDD of 0.35 mg/cm^2^, and Pu/PL of 1/1 and different Wt is demonstrated in Figure 12. It is worth noting that when Wt increases, the x_2_ and x_6_ fall while the x_1_, x_3_, x_4_, and x_5_ increase. For example, under SDD of 0.2 mg/cm^2^, NSDD of 0.35 mg/cm^2^, and P_u_/P_L_ of 1/1, the x_1_ increased by 13.4% and 15.4% when Wt increased from 3 to 6 mL/h and from 3 to 9 mL/h, respectively, whereas under the same conditions, the x_2_ decreased by 72.1% and 57.2% when Wt increased from 3 to 6 mL/h and from 6 to 9 mL/h, respectively.

#### 5.2.4. Indicator Trends under Different Nonuniform Pollution Distribution (P_u_/P_L_)

**Figure 13:**
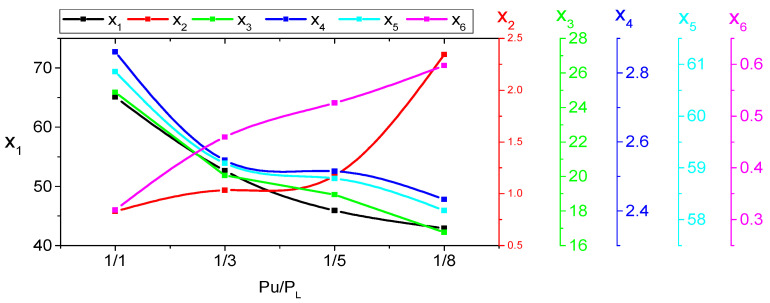
The impact of P_u_/P_L_ on indicator shift.

The relationship between proposed indices x_1_–x_6_ and nonuniform pollution distribution Pu/PL for a polluted porcelain insulator under SDD of 0.2 mg/cm^2^, NSDD of 0.35 mg/cm^2^, and Wt of 9 mL/h and different Pu/PL as an example is shown in Figure 13. It can be observed that an increase in Pu/PL causes an increase in the x_2_ and x_6_ and a decrease in the x_1_, x_3_, x_4_, and x_5_. This means that the sample under uniform contamination conditions is more dangerous in terms of flashover incidence than the sample under nonuniform pollution levels.

### 5.3. Insulator Condition Based on the Test Data of Indices

#### 5.3.1. Insulator Condition Classification Based on Test Preparation

In this section, the ranges of the indicators corresponded to the level of SDD, NSDD, Wt, and P_u_/P_L_ are classified. The experimental data indicated that the values of x_1_, x_3_, x_4_, and x_5_ increased in proportion to an increase in SDD, NSDD, and Wt, but a decrease in P_u_/P_L_. Meanwhile, the indicators x_2_ and x_6_ decreased with an increase in SDD, NSDD, and Wt, and decrease in P_u_/P_L_. The proposed index values in the normal range were observed under the clean and low-pollution cases with Wt less than 4 mL/h and NSDD less than 0.2 mg/cm^2^. In this case, the possibility of discharge occurrence is almost nonexistent. According to indicator results in Table 5, the insulator was in an abnormal state under low contamination (0.05 mg/cm^2^) with heavy wetting Wt (9 mL/h) and medium and high NSDD (0.25 and 0.35 mg/cm^2^) for all contamination distribution (Pu/PL), except when P_u_/P_L_ = 1/8. In addition, the insulator under examination displayed an abnormal condition in the presence of moderate pollution (0.12 mg/cm^2^) under moderate wetting Wt of 6 mL/h, NSDD of 0.25 mg/cm^2^, and P_u_/P_L_ of 1/1 and 1/5. The probability of a discharge occurring in these conditions is low, except in cases of extreme wetting, where the possibility of flashover increases. Meanwhile, the critical condition of the insulator under test was found under two circumstances: first, under medium contamination conditions with Wt of 9 mL/h, NSDD of 0.35 mg/cm^2^, and all contamination distribution P_u_/P_L_ cases; and second, when SDD is high under medium and heavy levels for Wt, NSDD, and all P_u_/P_L_ cases. The flashover possibility occurring in these conditions is high, especially under high wetting and high NSDD.

#### 5.3.2. Insulator Condition Classification Based on Proposed Indicators

**Figure 14:**
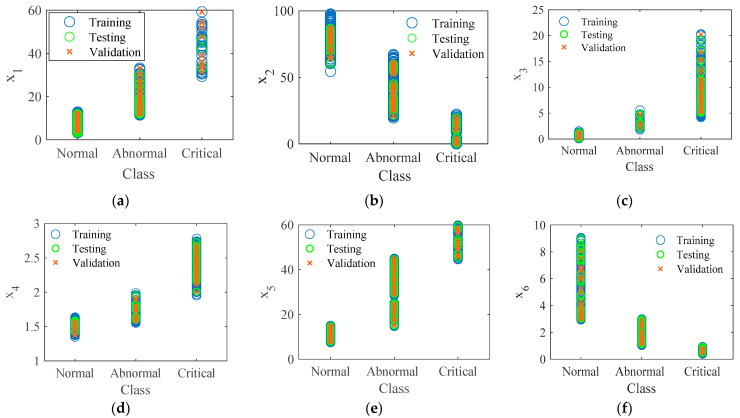
Visualization of the training samples of leakage current indicators: (**a**) x_1_; (**b**) x_2_; (**c**) x_3_; (**d**) x_4_; (**e**) x_5_; (**f**) x_6_.

To develop a statistical technique for identifying diagnostic indicator borders based on the x_1_, x_2_, x_3_, x_4_, x_5_, and x_6_ inputs, the naïve Bayes classification algorithm [46] was trained with the experimental data to predict the insulator’s state. In this study, MATLAB’s Deep Learning Toolbox was employed to develop the classification model. In the classification procedure, 952 data sets for each indicator were used in the classification process using the naïve Bayes classifier algorithm, where 70% of the data (666 data) were chosen for the model training, 15% of the data (143 data) were utilized for the model performance verification, and the other 15% of the data (143 data) were selected for the model testing, as shown in Figure 14. The boundary classification results of indicators x_1_, x_2_, x_3_, x_4_, x_5_, and x_6_ based on naïve Bayes classification are also shown in Figure 14.

**Figure 15:**
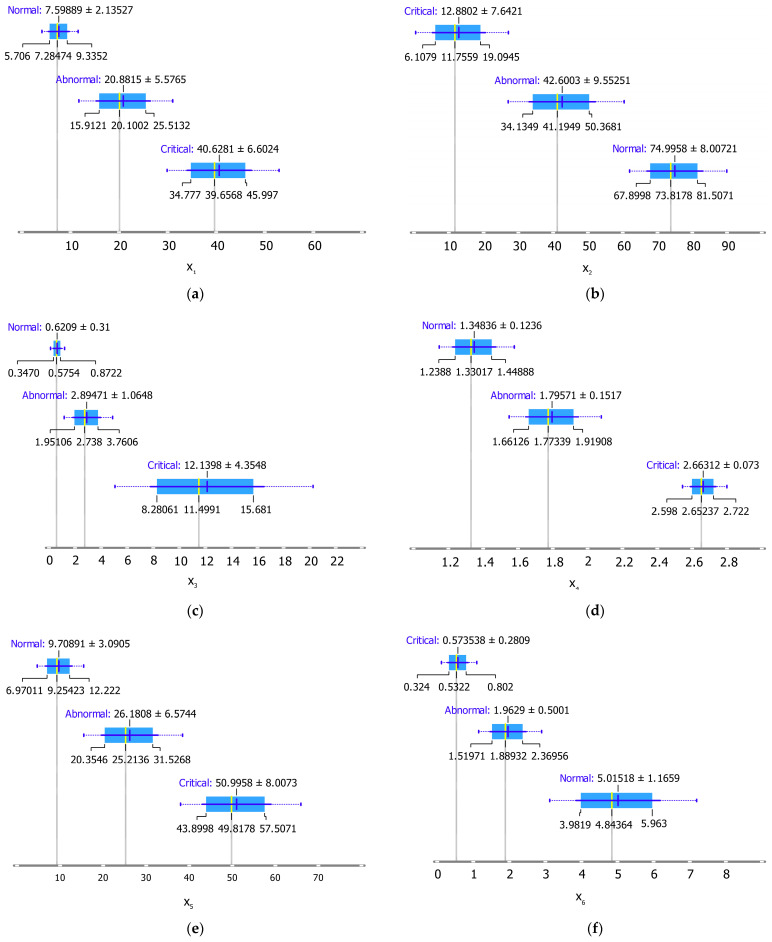
Limitation of insulator condition indicators: (**a**) x_1_; (**b**) x_2_; (**c**) x_3_; (**d**) x_4_; (**e**) x_5_; (**f**) x_6_.

Figure 15 depicts the limitations of the suggested indicators for diagnosing insulator conditions (normal, abnormal, and critical). Taking indicator x_1_ as an example, the insulator condition is normal when the x_1_ value is less than 7.59 mA, abnormal when it is about 20.88 mA, and critical when it is more than 46.6 mA. Of note, the restriction was set at 75% of each level’s data in the box plot (Figure 14). Therefore, the insulator state is normal when x_1_ is less than 9.33 and abnormal when x_1_ is between 15.9 mA and 25.3 mA; otherwise, the insulator condition is critical.

### 5.4. Determination of Indices’ Performance

**Figure 16:**
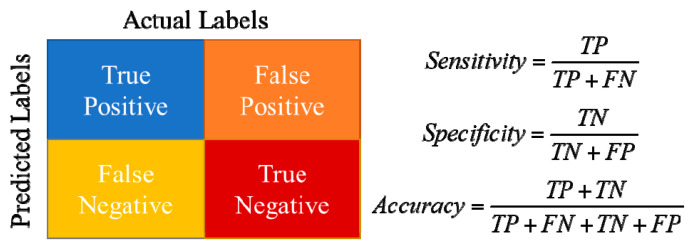
Confusion matrix for evaluating of the proposed indicators.

To determine the performance of the proposed indices precisely, the ability of the indicators to accurately estimate the insulator state taken from the data set of 952 tests observation was examined. The sensitivity, precision, and accuracy of these indices were calculated using the confusion matrix shown in Figure 16 during the assessment based on the naïve Bayes classification. The parameters of the confusion matrix were specified based on the insulator condition preparation. The decision of test results and proposed indices are defined as:

1. True positive (TP) represents the number of insulator tests that have been properly classified using the indicators to have pollution, meaning they have the pollution.
2. True negative (TN) represents the number of correctly classified insulator tests using the indicators as clean and it in fact is clean.
3. False positive (FP) represents the number of misclassified insulator tests using the indicators as polluted, but in fact they are polluted.
4. False negative (FN) represents the number of insulator tests misclassified using the indicators as polluted, but in fact are not polluted.

Experimentally, 905 test results out of 952 total tests reflected the insulator condition correctly, whereas 47 test results provided negative results. The explanation for the erroneous test results is a lack of implementation of pollutants on the insulator surface in a proper way or measuring-device error. In comparison to the total number of test outcomes, the number of prediction results for each indicator varied.

The variation in the prediction results may assist in determining which indicators are the best. It can be observed that the indices with a high number of correct prediction results are the best and will achieve high accuracy.

According to the indicator-prediction output, the greatest number of correct predicted results was observed for indicator x_6_ (906), followed by x_3_ (894), x_4_ (890), x_1_ (883), x_5_ (879), and x_2_ (873). As a result, the highest indicator accuracy is x_6_, followed by x_3_, x_4_, x_1_, x_5_, and x_2_ accordingly, as shown in Table 6. Meanwhile, the greatest sensitivity was in indicator x_3_, followed by x_6_, x_4_, x_1_, x_5_, and x_2_ accordingly. Moreover, the “best” performance is that where the variance between sensitivity and specificity is the least [47]. According to Table 5, the smallest difference between sensitivity and specificity is shown with the indicator x_6_, followed by x_3_, x_4_, x_1_, x_5_ and x_2_ accordingly. The sensitivity, precision, and accuracy of these indices were calculated using the confusion matrix shown in Figure 16 during the assessment.

## 6. Conclusions

This paper reports on an experimental investigation of leakage current indices to determine the stability of polluted insulators. Porcelain insulators of 11 kV and 33 kV distribution lines under different levels of contamination (SDD), wetting rate (Wt), insoluble deposit density (NSDD), and nonuniform distribution pollution (Pu/PL) were investigated. The temporal and frequency characteristics of the leakage current were then derived from the laboratory test results and used as assessment indicators for the insulators’ physical properties. The leakage current indication ranges were classified using the naïve Bayes technique into three levels of insulator health status. The suggested indicator’s performance was evaluated using the confusion matrix approach. The indicators used in this study are all useful in detecting the pollution levels of the insulators. According to the confusion matrix study, the odd harmonic indicator x_6_, slope indicator x_3_, and crest factor indicator x_4_ perform better than the others in terms of accuracy, with 0.918, 0.901, and 0.892, respectively.

## Figures and Tables

**Table 1:** Test insulator structure characteristics.

Parameter	Symbol	Length (cm)
Creepage distance	L	32
Insulator height	H	14.6
Insulator diameter	D	25.5
Rib diameters	*d*_1_	19.5
	*d*_2_	14.5
	*d*_3_	10.5
	*d_c_*	5

**Table 2:** Pollution layer characteristics.

σ_20_ (S/m)	SDD (mg/cm^2^)	NSDD (mg/cm^2^)	Wt (mL/h)	Pollution Level
0	0	0	0	Clean
0.39	0.05	0.15	3	Low
0.72	0.1	0.25	6	Medium
1.38	0.2	0.35	9	High

**Table 3:** LC characteristics for insulators under different contamination degree for uniform distribution.

			Single Insulator under 11 kV							3 Unit Insulator String under 33kV						
SDD	NSDD	Wt	Im	3rd	5th	7th	9th	THD	ϕ	Im	3rd	5th	7th	9th	THD	ϕ
0.00	0	0	1.807	0.005	0.020	0.012	0.020	7.727	≈90	1.778	0.005	0.019	0.012	0.019	8.523	≈ 90
	0.15	0	1.975	0.006	0.030	0.010	0.005	7.795	≈90	1.944	0.006	0.029	0.010	0.005	8.598	86.370
		3	4.344	0.030	0.149	0.020	0.008	8.492	87.026	4.276	0.029	0.147	0.019	0.008	9.367	82.100
		6	7.109	0.039	0.125	0.079	0.007	8.595	86.956	6.997	0.039	0.123	0.078	0.007	9.481	82.034
		9	8.886	0.069	0.168	0.099	0.059	9.190	85.838	8.746	0.068	0.165	0.097	0.058	10.136	80.980
	0.25	0	2.172	0.030	0.138	0.020	0.020	7.944	88.802	2.138	0.029	0.136	0.019	0.019	8.762	83.776
		3	6.615	0.099	0.405	0.049	0.059	9.064	87.076	6.511	0.097	0.398	0.049	0.058	9.998	82.147
		6	8.590	0.107	0.395	0.079	0.069	9.133	87.166	8.454	0.105	0.389	0.078	0.068	10.073	82.232
		9	9.577	0.168	0.592	0.099	0.079	9.578	86.277	9.426	0.165	0.583	0.097	0.078	10.565	81.394
	0.35	0	4.048	0.057	0.118	0.089	0.089	8.355	88.084	3.984	0.056	0.117	0.087	0.087	9.216	83.098
		3	7.306	0.178	0.592	0.049	0.030	9.601	85.888	7.191	0.175	0.583	0.049	0.029	10.590	81.027
		6	8.984	0.197	0.627	0.059	0.049	9.853	85.269	8.843	0.194	0.617	0.058	0.049	10.867	80.443
		9	10.367	0.326	0.721	0.069	0.089	9.978	84.870	10.203	0.321	0.709	0.068	0.087	11.006	80.066
0.05	0.15	0	5.134	0.089	0.425	0.089	0.039	9.384	82.036	5.053	0.087	0.418	0.087	0.039	10.351	77.392
		3	8.886	1.234	3.159	0.592	0.296	11.693	79.152	8.746	1.215	3.110	0.583	0.292	12.897	74.671
		6	11.749	1.343	2.962	0.819	0.395	12.904	67.365	11.564	1.322	2.915	0.807	0.389	14.234	63.552
		9	15.501	1.777	3.554	0.908	0.760	16.482	57.495	15.257	1.749	3.498	0.894	0.748	18.180	54.241
	0.25	0	7.207	0.138	0.553	0.079	0.069	10.174	80.589	7.094	0.136	0.544	0.078	0.068	11.222	76.027
		3	12.144	1.402	2.281	0.839	0.652	12.447	66.078	11.953	1.380	2.245	0.826	0.641	13.729	62.338
		6	14.711	1.649	2.271	1.382	0.691	14.688	54.142	14.479	1.623	2.235	1.360	0.680	16.200	51.077
		9	18.166	1.767	2.281	1.481	0.790	17.179	42.575	17.880	1.739	2.245	1.458	0.777	18.949	40.165
	0.35	0	8.984	0.178	0.642	0.128	0.099	11.008	78.164	8.843	0.175	0.632	0.126	0.097	12.142	73.739
		3	16.093	1.876	2.488	1.007	0.553	15.248	56.098	15.840	1.846	2.449	0.991	0.544	16.818	52.922
		6	20.141	2.172	2.370	1.382	0.889	18.025	40.050	19.824	2.138	2.332	1.360	0.875	19.882	37.783
		9	25.769	2.577	3.159	1.185	1.086	21.534	33.154	25.363	2.536	3.110	1.166	1.069	23.752	31.277
0.1	0.15	0	6.319	0.217	0.711	0.109	0.079	9.384	71.058	6.553	0.225	0.737	0.113	0.082	10.444	68.988
		3	14.612	3.258	2.666	0.395	0.790	17.522	55.100	15.153	3.379	2.764	0.410	0.819	19.502	53.495
		6	16.093	4.048	3.159	0.612	1.185	21.580	38.453	16.688	4.198	3.276	0.635	1.229	24.018	37.333
		9	19.055	4.542	4.048	0.938	0.790	26.575	27.106	19.760	4.710	4.198	0.973	0.819	29.578	26.316
	0.25	0	7.405	0.316	0.790	0.128	0.207	11.293	75.739	7.679	0.328	0.819	0.133	0.215	12.569	73.533
		3	16.982	3.456	3.061	1.027	0.889	24.849	52.415	17.610	3.583	3.174	1.065	0.921	27.657	50.889
		6	19.351	5.233	3.653	1.086	0.968	28.861	30.160	20.067	5.426	3.788	1.126	1.003	32.122	29.281
		9	23.103	5.628	4.048	1.283	1.086	36.542	25.070	23.958	5.836	4.198	1.331	1.126	40.671	24.340
	0.35	0	9.281	0.405	0.790	0.306	0.227	11.327	70.289	9.624	0.420	0.819	0.317	0.235	12.607	68.242
		3	19.549	5.529	3.554	1.283	1.580	34.873	39.581	20.272	5.733	3.686	1.331	1.638	38.814	38.428
		6	20.141	7.109	4.048	1.481	1.283	39.582	20.589	20.886	7.372	4.198	1.536	1.331	44.055	19.989
		9	25.769	6.121	4.147	1.678	1.777	48.052	14.920	26.722	6.348	4.300	1.741	1.843	53.482	14.486
0.2	0.15	0	8.313	0.523	0.948	0.444	0.316	11.339	56.727	8.621	0.543	0.983	0.461	0.328	12.620	55.074
		3	26.065	9.478	2.764	2.073	1.481	41.788	32.056	27.029	9.829	2.867	2.150	1.536	46.510	31.122
		6	29.027	9.774	2.271	2.567	1.086	47.229	17.545	30.101	10.136	2.355	2.662	1.126	52.566	17.034
		9	36.036	10.732	2.271	2.666	1.283	50.029	11.537	37.370	11.129	2.355	2.764	1.331	55.682	11.201
	0.25	0	10.169	0.612	0.987	0.622	0.316	12.322	42.455	10.545	0.635	1.024	0.645	0.328	13.714	41.219
		3	28.928	9.379	2.764	2.073	1.086	52.155	26.397	29.998	9.726	2.867	2.150	1.126	58.049	25.628
		6	32.482	10.762	2.666	2.271	1.185	54.681	10.709	33.684	11.160	2.764	2.355	1.229	60.860	10.397
		9	39.393	12.835	3.159	2.370	1.185	61.482	1.038	40.851	13.310	3.276	2.457	1.229	68.429	1.008
	0.35	0	10.564	0.642	1.007	0.474	0.494	12.150	32.774	10.955	0.665	1.044	0.491	0.512	13.523	31.820
		3	34.950	13.743	2.962	1.185	1.481	59.059	3.912	36.244	14.252	3.071	1.229	1.536	65.732	3.798
		6	51.735	15.994	3.456	2.073	1.185	67.768	2.066	53.649	16.586	3.583	2.150	1.229	75.426	2.006
		9	64.273	17.673	2.666	1.283	1.678	69.563	0.828	66.651	18.327	2.764	1.331	1.741	77.424	0.804

**Table 4:** LC indices of nonuniformly polluted insulators under different Wt and NSDD for 11 kV insulators.

Pu/PL			1/3						1/5						1/8					
SDD mg/cm^2^	NSDD mg/cm^2^	Wt mL/h	x_1_	x_2_	x_3_	x_4_	x_5_	x_6_	x_1_	x_2_	x_3_	x_4_	x_5_	x_6_	x_1_	x_2_	x_3_	x_4_	x_5_	x_6_
0.05	0.15	0	4.2	84.67	0.14	1.43	7.97	6.96	3.7	86.36	0.13	1.43	7.93	8.42	3.4	86.62	0.13	1.42	7.85	8.99
		3	7.3	81.69	0.37	1.52	9.93	3.42	6.3	83.32	0.36	1.52	9.88	3.53	5.9	83.57	0.36	1.51	9.78	3.72
		6	9.6	69.53	0.78	1.56	10.96	3.18	8.4	70.92	0.77	1.55	10.91	3.35	7.8	71.13	0.77	1.55	10.79	3.51
		9	12.7	59.34	1.03	1.57	14.00	3.04	11.1	60.53	1.02	1.57	13.93	3.07	10.3	60.71	1.01	1.56	13.78	3.45
	0.25	0	6	83.17	0.30	1.58	8.64	6.84	5.2	84.84	0.30	1.58	8.60	7.88	4.9	85.09	0.30	1.57	8.51	8.22
		3	10.1	68.20	0.69	1.55	10.57	2.82	8.8	69.56	0.69	1.55	10.52	2.96	8.2	69.77	0.68	1.54	10.41	3.08
		6	12.2	55.88	1.07	1.56	12.48	2.69	10.7	57.00	1.06	1.56	12.41	2.80	10	57.17	1.05	1.55	12.28	3.02
		9	15.1	43.94	3.09	1.58	14.59	2.67	13.2	44.82	3.07	1.57	14.52	2.83	12.3	44.95	3.04	1.57	14.37	2.83
	0.35	0	7.4	80.67	0.52	1.59	9.35	6.25	6.5	82.28	0.52	1.59	9.30	7.42	6	82.53	0.51	1.58	9.21	8.30
		3	13.3	57.90	0.87	1.56	12.95	2.18	11.6	59.05	0.87	1.55	12.89	2.36	10.8	59.23	0.86	1.55	12.75	2.48
		6	16.6	41.33	3.03	1.57	15.31	2.20	14.5	42.16	3.02	1.57	15.23	2.41	13.5	42.29	2.99	1.56	15.08	2.53
		9	21.3	34.22	5.63	1.58	18.29	2.12	18.5	34.90	5.60	1.58	18.20	2.30	17.3	35.01	5.55	1.58	18.01	2.40
0.1	0.15	0	5.1	73.33	0.63	1.60	7.97	5.34	4.5	74.80	0.62	1.60	7.93	6.29	4.2	75.03	0.62	1.59	7.85	7.88
		3	12	56.87	2.25	1.53	14.88	1.30	10.5	58.00	2.24	1.53	14.81	1.62	9.8	58.18	2.22	1.52	14.65	1.95
		6	13.3	39.69	4.69	1.59	18.33	1.33	11.6	40.48	4.67	1.58	18.24	1.50	10.8	40.60	4.62	1.58	18.05	1.68
		9	15.7	27.97	8.09	2.22	22.57	1.48	13.7	28.53	8.04	2.22	22.46	1.53	12.8	28.62	7.97	2.21	22.23	1.71
	0.25	0	5.9	78.17	0.78	2.42	9.59	5.06	5.1	79.73	0.78	2.42	9.54	5.73	4.8	79.97	0.77	2.41	9.44	7.35
		3	13.5	54.10	5.63	1.60	21.11	1.44	11.8	55.18	5.60	1.60	21.00	1.56	11	55.34	5.55	1.59	20.78	1.64
		6	15.4	31.13	6.75	2.17	24.51	1.31	13.4	31.75	6.71	2.17	24.39	1.48	12.5	31.84	6.65	2.16	24.14	1.55
		9	17.5	25.87	8.58	2.41	31.04	1.31	15.3	26.39	8.53	2.41	30.89	1.56	14.3	26.47	8.45	2.40	30.56	1.60
	0.35	0	7.6	72.54	0.79	2.58	9.62	4.16	6.6	73.99	0.79	2.58	9.57	4.70	6.2	74.22	0.78	2.57	9.47	5.90
		3	16	40.85	5.15	1.62	29.62	1.16	14	41.67	5.13	1.61	29.47	1.37	13	41.79	5.08	1.61	29.17	1.45
		6	16.5	21.25	7.35	2.21	33.62	1.16	14.4	21.67	7.31	2.21	33.45	1.28	13.4	21.74	7.24	2.20	33.10	1.52
		9	21.1	15.40	10.35	2.48	40.82	1.20	18.4	15.71	10.29	2.48	40.61	1.23	17.2	15.75	10.20	2.47	40.19	1.41
0.2	0.15	0	6.8	58.55	1.03	2.65	9.63	3.42	5.7	59.72	1.02	2.64	9.58	3.78	5.4	59.90	1.01	2.63	9.48	4.72
		3	21.4	33.08	5.88	1.76	35.50	0.67	18.6	33.75	5.85	1.76	35.32	0.76	17.4	33.85	5.79	1.76	34.95	0.91
		6	23.8	18.11	7.63	2.38	40.12	0.63	20.7	18.47	7.58	2.38	39.92	0.66	19.4	18.52	7.51	2.37	39.50	0.79
		9	29.5	11.91	11.27	2.51	42.50	0.60	25.7	12.14	11.21	2.51	42.28	0.57	24.1	12.18	11.10	2.50	41.84	0.72
	0.25	0	8.3	43.82	1.26	2.70	10.47	3.28	7.3	44.69	1.26	2.69	10.41	3.58	6.8	44.83	1.24	2.68	10.30	4.31
		3	23.7	27.24	9.56	1.82	44.30	0.71	20.7	27.79	9.51	1.81	44.08	0.72	19.3	27.87	9.42	1.81	43.62	0.76
		6	26.6	11.05	11.17	2.46	46.45	0.63	23.2	11.27	11.10	2.45	46.22	0.71	21.7	11.31	11.00	2.44	45.73	0.71
		9	32.3	1.07	15.05	2.47	52.22	0.54	28.1	1.09	14.96	2.47	51.96	0.58	26.3	1.10	14.82	2.46	51.42	0.65
	0.35	0	8.7	33.83	0.63	2.63	10.32	3.21	7.5	34.50	0.63	2.63	10.27	3.48	7.1	34.61	0.62	2.61	10.16	4.05
		3	30.5	4.04	10.36	1.93	50.17	0.56	27.9	4.12	10.30	1.92	49.92	0.64	16.7	4.13	10.20	1.92	49.39	0.78
		6	46.2	2.13	12.77	2.49	57.56	0.67	32.9	2.17	12.70	2.49	57.28	0.57	21.4	2.18	12.58	2.48	56.68	0.67
		9	52.7	0.00	20.06	2.55	59.09	0.46	45.9	0.00	18.95	2.54	58.79	0.53	42.9	0.00	16.76	2.53	58.18	0.60

**Table 5:** LC indices of nonuniformly polluted insulators under different Wt and NSDD for insulator string 33 kV.

Pu/PL			1/3						1/5						1/8					
SDD mg/cm^2^	NSDD mg/cm^2^	Wt mL/h	x_1_	x_2_	x_3_	x_4_	x_5_	x_6_	x_1_	x_2_	x_3_	x_4_	x_5_	x_6_	x_1_	x_2_	x_3_	x_4_	x_5_	x_6_
0.05	0.15	0	4	79.80	0.15	1.47	8.18	7.21	3.5	81.39	0.14	1.47	8.14	8.72	3.3	81.64	0.14	1.46	8.05	9.31
		3	7	76.99	0.39	1.57	10.19	3.54	6	78.53	0.38	1.57	10.14	3.66	5.7	78.77	0.38	1.56	10.03	3.85
		6	9.2	65.53	0.83	1.61	11.24	3.29	8.1	66.84	0.82	1.60	11.19	3.47	7.5	67.04	0.82	1.60	11.07	3.64
		9	12.2	55.93	1.09	1.62	14.36	3.15	10.6	57.05	1.08	1.62	14.29	3.18	9.9	57.22	1.07	1.61	14.14	3.57
	0.25	0	5.8	78.39	0.32	1.63	8.86	7.09	5	79.96	0.32	1.63	8.82	8.16	4.7	80.20	0.32	1.62	8.73	8.52
		3	9.7	64.28	0.73	1.60	10.84	2.92	8.4	65.56	0.73	1.60	10.79	3.07	7.9	65.76	0.72	1.59	10.68	3.19
		6	11.7	52.67	1.14	1.61	12.80	2.79	10.3	53.72	1.12	1.61	12.73	2.90	9.6	53.88	1.11	1.60	12.60	3.13
		9	14.5	41.41	3.28	1.63	14.97	2.77	12.7	42.24	3.26	1.62	14.90	2.93	11.8	42.37	3.23	1.62	14.74	2.93
	0.35	0	7.1	76.03	0.55	1.64	9.59	6.48	6.2	77.55	0.55	1.64	9.54	7.69	5.8	77.79	0.54	1.63	9.45	8.60
		3	12.8	54.57	0.92	1.61	13.29	2.26	11.1	55.66	0.92	1.60	13.23	2.44	10.4	55.82	0.91	1.60	13.08	2.57
		6	15.9	38.95	3.21	1.62	15.71	2.28	13.9	39.74	3.20	1.62	15.63	2.50	12.9	39.86	3.17	1.61	15.47	2.62
		9	20.4	32.25	5.97	1.63	18.77	2.20	17.7	32.89	5.94	1.63	18.67	2.38	16.6	33.00	5.89	1.63	18.48	2.49
0.12	0.15	0	4.9	69.11	0.67	1.65	8.18	5.53	4.3	70.50	0.66	1.65	8.14	6.52	4	70.72	0.66	1.64	8.05	8.16
		3	11.5	53.60	2.39	1.58	15.27	1.35	10.1	54.67	2.38	1.58	15.20	1.68	9.4	54.84	2.36	1.57	15.03	2.02
		6	12.8	37.41	4.98	1.64	18.81	1.38	11.1	38.15	4.95	1.63	18.71	1.55	10.4	38.27	4.90	1.63	18.52	1.74
		9	15.1	26.36	8.58	2.29	23.16	1.53	13.1	26.89	8.53	2.29	23.04	1.59	12.3	26.97	8.46	2.28	22.81	1.77
	0.25	0	5.7	73.68	0.83	2.50	9.84	5.24	4.9	75.15	0.83	2.50	9.79	5.94	4.6	75.37	0.82	2.48	9.69	7.61
		3	12.9	50.99	5.97	1.65	21.66	1.49	11.3	52.01	5.94	1.65	21.55	1.62	10.5	52.16	5.89	1.64	21.32	1.70
		6	14.8	29.34	7.16	2.24	25.15	1.36	12.8	29.92	7.12	2.24	25.02	1.53	12	30.01	7.06	2.23	24.77	1.61
		9	16.8	24.38	9.10	2.48	31.85	1.36	14.7	24.87	9.05	2.48	31.69	1.62	13.7	24.95	8.97	2.47	31.35	1.66
	0.35	0	7.3	68.37	0.84	2.66	9.87	4.31	6.3	69.74	0.84	2.66	9.82	4.87	5.9	69.95	0.83	2.65	9.72	6.11
		3	15.3	38.50	5.46	1.67	30.39	1.20	13.4	39.27	5.44	1.66	30.24	1.42	12.5	39.39	5.39	1.66	29.93	1.50
		6	15.8	20.03	7.80	2.28	34.49	1.20	13.8	20.42	7.76	2.28	34.32	1.33	12.8	20.49	7.68	2.27	33.96	1.57
		9	20.2	14.51	10.98	2.56	41.88	1.24	17.6	14.81	10.92	2.56	41.67	1.27	16.5	14.84	10.82	2.55	41.23	1.46
0.2	0.15	0	6.5	55.18	1.09	2.73	9.88	3.54	5.5	56.29	1.08	2.72	9.83	3.92	5.2	56.46	1.07	2.71	9.73	4.89
		3	20.5	31.18	6.24	1.81	36.42	0.69	17.8	31.81	6.21	1.81	36.24	0.79	16.7	31.90	6.14	1.81	35.86	0.94
		6	22.8	17.07	8.10	2.45	41.16	0.65	19.8	17.41	8.04	2.45	40.96	0.68	18.6	17.46	7.97	2.44	40.53	0.82
		9	28.3	11.23	11.96	2.59	43.61	0.62	24.6	11.44	11.89	2.59	43.38	0.59	23.1	11.48	11.78	2.58	42.93	0.75
	0.25	0	8	41.30	1.34	2.78	10.74	3.40	7	42.12	1.34	2.77	10.68	3.71	6.5	42.25	1.32	2.76	10.57	4.47
		3	22.7	25.67	10.14	1.88	45.45	0.74	19.8	26.19	10.09	1.87	45.23	0.75	18.5	26.27	9.99	1.87	44.75	0.79
		6	25.5	10.41	11.85	2.54	47.66	0.65	22.2	10.62	11.78	2.53	47.42	0.74	20.8	10.66	11.67	2.52	46.92	0.74
		9	31	1.01	15.97	2.55	53.58	0.56	26.9	1.03	15.87	2.55	53.31	0.60	25.2	1.04	15.72	2.54	52.76	0.67
	0.35	0	8.3	31.89	0.67	2.71	10.59	3.33	7.2	32.52	0.67	2.71	10.54	3.61	6.8	32.62	0.66	2.69	10.42	4.20
		3	29.2	3.81	10.99	1.99	51.47	0.58	26.7	3.88	10.93	1.98	51.22	0.66	16	3.89	10.82	1.98	50.67	0.81
		6	44.3	2.01	13.55	2.57	59.06	0.69	31.5	2.05	13.47	2.57	58.77	0.59	20.5	2.05	13.35	2.56	58.15	0.69
		9	50.5	0.79	21.28	2.63	60.63	0.48	44	0.32	20.11	2.62	60.32	0.55	41.1	0.56	17.78	2.61	59.69	0.62

**Table 6:** The indices’ sensitivity, specificity, and accuracy for the 143 tests based on naïve Bayes algorithm.

Indicator	Sensitivity	Specificity	Accuracy
x_1_	0.932	0.329	0.868
x_2_	0.947	0.295	0.849
x_3_	0.951	0.452	0.901
x_4_	0.948	0.413	0.892
x_5_	0.950	0.338	0.868
x_6_	0.954	0.540	0.918
